# Driving Spray Drying towards Better Yield: Tackling a Problem That Sticks Around

**DOI:** 10.3390/pharmaceutics15082137

**Published:** 2023-08-14

**Authors:** Pavithra Sundararajan, Justin Moser, Lisa Williams, Tiffany Chiang, Colin Riordan, Matthew Metzger, Fan Zhang-Plasket, Fang Wang, John Collins, Joseph Williams

**Affiliations:** 1 Merck & Co., Inc., Rahway, NJ 07065, USA; matthew.metzger@merck.com (M.M.); fang.wang6@merck.com (F.W.); 2 Merck & Co., Inc., West Point, PA 19486, USA; justin_moser@merck.com (J.M.); tiffany.chiang@merck.com (T.C.); colin.riordan@merck.com (C.R.); fan_zhang-plasket@merck.com (F.Z.-P.); 3 MSD, Ballydine, E91 V091 Clonmel, Ireland; lisa.williams@merck.com (L.W.); john.collins@merck.com (J.C.); joe.williams@merck.com (J.W.)

**Keywords:** wall deposition, glass transition temperature, sorption kinetics, process product and performance, start-up and shutdown operation

## Abstract

Powder deposition and accumulation on walls of spray drying chamber has been known to impact spray drying processes, resulting in lower yield, frequent shutdowns, and downtimes. Critical factors that impact the extent and rate of wall deposition have been studied extensively in the chemical and food industry. In this paper, we present an atypical process yield issue wherein acceptable yield is obtained during the first batch of spray drying but undergoes significant yield loss in consecutive batches. Through understanding the interplay of the process, material properties, and equipment, we identify key mechanisms that are playing a role in causing the process yield issue. These mechanisms include surface roughness of the inner wall of the spray dryer, variation in gas flow due to the introduction of process analytical technology, start-up and shutdown operating parameters that expose the wall deposited powder from the prior batch to temperatures close to the onset of glass transition temperature and cause depression of its glass transition temperature. These factors result in more wall accumulation and impact the yield in subsequent batches. By correcting for most of these factors, the yield reduction issue was mitigated, and processing efficiency was improved.

## 1. Introduction

Spray drying is commonly used commercially across several industries to make spray-dried food products, chemicals, pharmaceutical drug products, and many more. Spray drying processes are used to remove liquid from a suspension, emulsion, or solution (feed) to generate dried solids, by spraying the feed into a hot drying medium [1]. During spray drying, some particles, often those with high liquid content could collect on the inner walls of the spray dryer, leading to the wall deposition problem [2]. It is well known, since the inception of spray drying, that wall deposition of powders resulting in powder caking can pose a challenge by reducing the yield of the spray-dried products. Yield is defined as the ratio of the wet spray-dried product collected from the cyclone to the total solids in the solution fed into the spray dryer. Yield reduction can result in a frequent shutdown of the spray dryer due to product loss and long downtimes for cleaning the dryers. Wall deposition could also affect the product quality if the powder on the wall is sensitive to prolonged time-temperature exposures during subsequent batch manufacture.

To understand the driving forces leading to wall deposition during spray drying, experimental studies and modeling [1,3,4,5,6] were conducted to assess the issue in the milk powder industry. Sticky-point temperature is defined as the temperature at which powdery material will start agglomerating due to the interplay of adhesive and cohesion forces [7]. The main adhesive forces acting on the particle are liquid bridging, Van der Waal forces, and electrostatic forces [8]. Liquid bridging force, if present, dominates over the others. The correlation between sticky point temperature, the glass transition temperature, and sorption properties has been investigated to understand and prevent sticking [7,9,10]. The interplay of material properties with processing parameters such as relative humidity or relative saturation (for non-aqueous solvents) and the relative difference between glass transition temperature and operating temperature in the dryer could impact the rate and extent of the wall deposition [11,12]. Differences in airflow patterns, such as varying swirl patterns and the spray cone angle, were also shown to have an effect on changing the extent of wall deposition [5,12,13,14]. Researchers in the food industry have leveraged the learnings to assess sticky-point temperature and understand the interplay with process parameters to overcome wall deposition during their at-scale manufacturing [15,16].

In the pharmaceutical industry, the spray drying process optimization studies often follow the formulation development studies. The process optimization experiments focus on designing wide enough process ranges such that there is no impact on the critical quality attributes such as stability, dissolution, etc., in addition to ensuring adequate process efficiency of the process. Significant attention in the pharmaceutical literature is focused on process aspects such as modeling and scale-up of the spray drying process [17,18,19] as well as fundamental aspects such as understanding single droplet drying with respect to changes in particle morphology [20] and predicting changes in glass transition temperature as a function of drying time [21]. However, there is limited literature on its application to the challenges of wall deposition risk and its impact on product and process performance, especially with a focus on at-scale demonstrations of minimization of wall deposition mitigation strategies. We note that while pharmaceutical spray drying is usually not conducted at the same scale as other industries such as chemicals or food, many of the root causes impacting wall deposition are not expected to be different from these industries. However, approaches to overcome the yield issue may differ depending on the constraints on process due to limitations from stability, dissolution or other performance attributes that are monitored against quality specifications.

In this paper, we present an experimental study involving a root cause investigation of an atypical yield behavior involving wall deposition during spray drying of a model compound on a pharmaceutical spray dryer. The spray drying process for the compound was established after confirming the acceptability of attributes of the spray-dried powder over a range of process parameters such as liquid-to-gas flow rates and process outlet temperature of the spray dryer. The spray drying process was established to operate below the glass transition temperature of the wet spray-dried powder. Atypical yield behavior was noticed when spray drying consecutive batches. The yield of the second batch was significantly lower whereas the yield of the first batch was high. Our results showcase an experimental solution built based on the understanding gained through the root cause investigation. The solution involved key process modifications that mitigated the yield issue, such as revising the process start-up and shutdown conditions that minimize the depression of the wet spray-dried powder’s glass transition temperature coupled with eliminating factors that contribute to gas flow variability. We conclude with a discussion focusing on the successes and limitations of the approach and recommendations for future products to address the yield challenge.

## 2. Experimental Section

### 2.1. Materials

Hydroxy propyl methyl cellulose acetate succinate (HPMCAS) was purchased from Shin-Etsu (Tokyo, Japan). The crystalline drug substance was supplied by Merck & Co., Inc., Rahway, NJ, USA. Acetone was purchased from Sasol Solvents (Secunda, South Africa).

### 2.2. Methods

**Figure 1:**
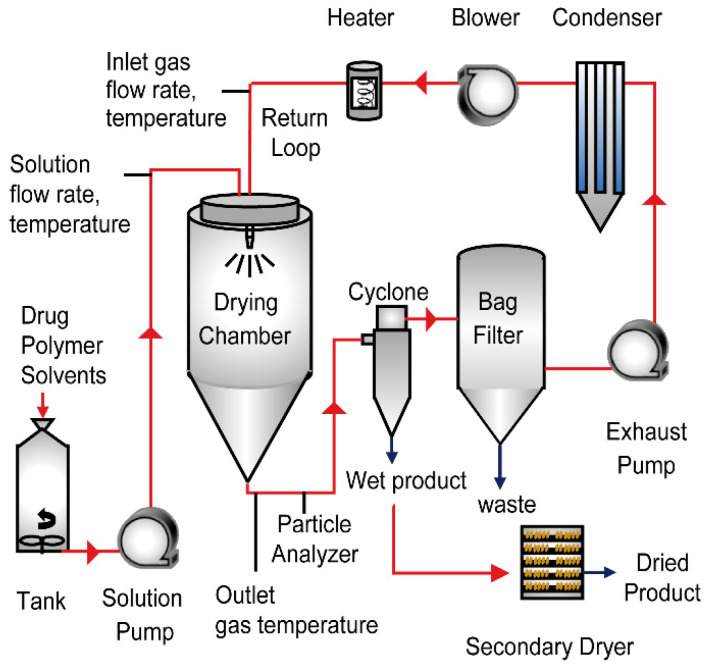
Schematic of Spray Dryer operating in a closed cycle.

The crystalline drug substance and HPMCAS are dissolved in acetone and the resulting solution was spray-dried on a co-current PharmaSD^®^ Type PSD-2 spray dryer (referred to as Dryer 1) modified to run at a nominal drying gas flow rate of 400 kg/h in a closed cycle, as depicted in the schematic in Figure 1 with all its components. The spray solution is pumped from the solution tank through the nozzle at a flow rate of 44 kg/h and sprayed inside the drying chamber. The drying gas used in the chamber is nitrogen which is heated and fed into the chamber using the gas disperser. The spray-dried powder leaves the dryer and is separated from the gas using a cyclone. Outlet temperature after the exit of the dryer is controlled to 43 °C. A particle analyzer is fitted in the connection between the spray dryer and cyclone to measure particle sizes in-situ. The gas leaving the cyclone contains nitrogen, evaporated solvent, and residual powder. The gas passes through a bag house filter to remove residual particles and then through a condenser (set at −15 °C) to remove the solvent. The nitrogen gas is reheated and pumped back into the spray dryer. Two batches are sprayed consecutively and the theoretical batch size of these batches is 60 kg of spray-dried powder. The resulting powder is further dried in a secondary drying process to reduce the acetone level below the desired ICH limits of 5000 ppm.

Prior to spray drying a given batch, a startup procedure is conducted. The drying chamber is heated using nitrogen gas to an inlet temperature of 100 °C for a minimum of 2 h. The condenser is set at 5 °C during this period. Next, acetone is sprayed at 39 kg/h with the condenser setting revised to −15 °C to reach the steady state outlet temperature of 43 °C. Thereafter, the feed solution is processed. At the end of a batch, a shutdown procedure is conducted. After the feed solution is processed, acetone is spray-dried at 39 kg/h for approximately 1 h. Thereafter the inlet process temperature is ramped down to room temperature.

A second spray drying experiment involving spray drying two consecutive batches (theoretical batch size of 72 kg each) of the same drug-polymer solution was also conducted on a second modified Pharma-SD^®^ Type PSD-2 dryer in a closed cycle of similar but not identical design (Dryer 2). The startup and shutdown conditions used on dryer 2 were at the same set points for temperature and flow rates but for a much shorter duration. An additional difference was that during the heat-up phase, the condenser was set at −15 °C and maintained throughout the run.

The methods used for the characterization of the spray-dried powders are provided in the supplementary information section.

## 3. Results and Discussion

### 3.1. Wall Deposition Problem

An atypical process yield behavior was observed during the spray-drying process development of the spray-dried powder of the model compound on dryer 1. The initial batch processed on a fully cleaned spray dryer achieved a wet yield of 98%. A typical acceptable wet yield for a spray-dried polymer product is >80% since some powder deposition on the wall is common and cannot be recovered. Additionally, wet yields above 100% can sometimes be observed because the drying solvent has not been completely removed from the powder. Most often, the spray drying process with yield issues shows the risk of yield loss in the first batch. Others might have a decreasing trend in yield in subsequent batches. However, in this experiment, the entire second batch was plagued with less than expected collection of spray dried powder with a significant yield reduction and net wet yield of 59% (Poor collection was noted through out the batch. To avoid significant wastage, in the middle of the second batch, solution spray was stopped and solvent was sprayed. An additional 15% of the batch was recovered. At the end of batch, additional gas flow was run to dislodge wall deposits and 13% of batch was recovered. 5% of material was recovered from wiping the walls of the chamber. These are not included in the calculation of the net wet yield in this paper.).

**Figure 2:**
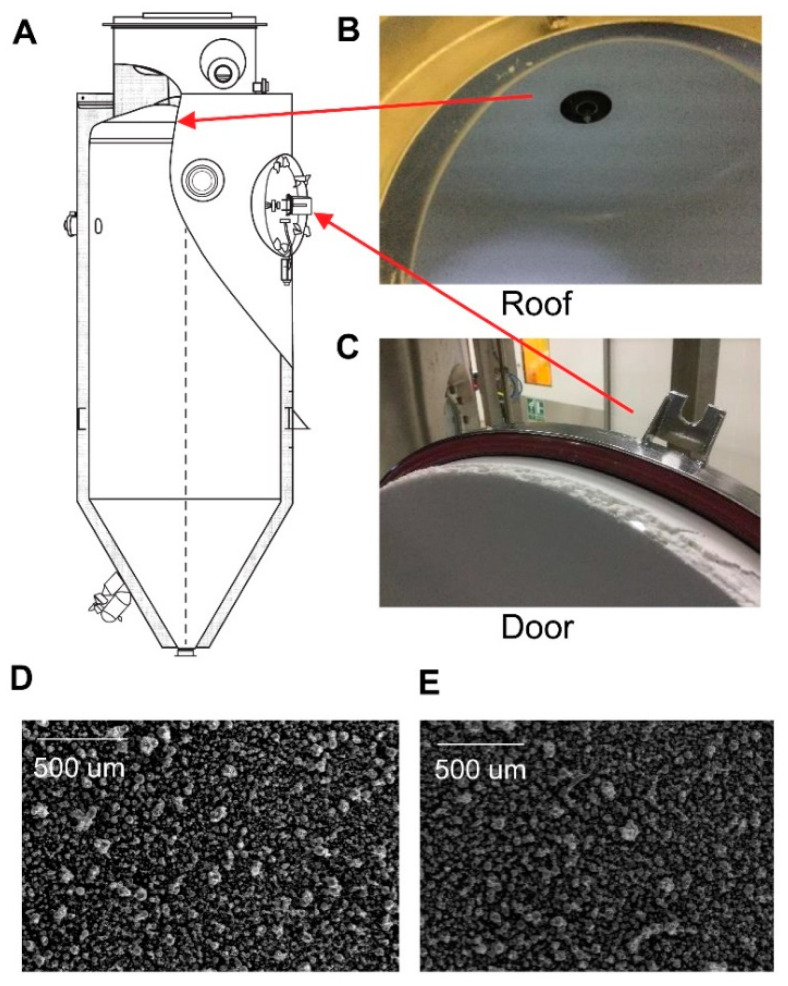
Wall Deposition after the atypical yield issue: (**A**) Schematic of spray dryer with focal points highlighted in red. Wall Deposition on the (**B**) roof and (**C**) door of the drying chamber. SEM of the powder collected during a steady-state run (**D**) and of the wall-deposited powder (**E**).

After the completion of the batch, the inner chamber walls were noted to have significant atypical powder deposition on the roof and near the top regions of the dryer as shown in Figure 2A–C.

Samples of the powder collected from the cyclone during steady state operation and wall deposited powder were characterized and confirmed to have similar properties as seen by the morphology SEM images in Figure 2D,E, particle size and density along with measured glass transition temperature, T_g_ in Table 1. The details of characterization techniques are included in the supplementary section. The differences in the material properties are within the standard batch-to-batch variability. These observations confirm that other common failure modes leading to spray dryer wall deposition, such as incomplete atomization or poor droplet drying leading to agglomeration on walls, were not contributing to this specific problem as significantly larger and agglomerated particles and associated potential shifts in bulk density properties particles would have been noted.

### 3.2. Approach to Evaluate Potential Root Causes

#### 3.2.1. Repeatability Test

At the outset of troubleshooting the atypical yield problem, we established an experiment to test the occurrence of yield reduction problems during two-batch manufacture on a second Pharma Spray Dryer-2. Same operating conditions, namely, same set of inlet conditions (inlet gas temperature, gas and solution flow rate, and condenser temperature) were used and these resulted in a similar outlet temperature at the exit of the spray dryer. However, the two spray dryers and their operation had some potential and some known differences, namely, (i) potential differences in the design of gas dispersers (proprietary knowledge), (ii) differences in specifications on the surface finish of inner walls, (iii) the second spray-dryer did not include the in-situ particle size analyzer, and (iv) differences in start-up and shut-down conditions.

**Figure 3:**
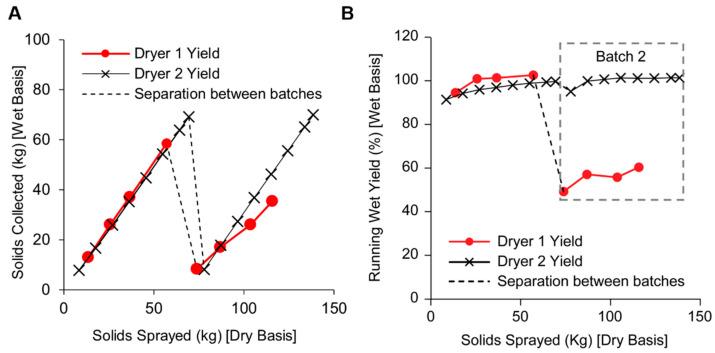
Comparison of solids collected (**A**) and running wet yield (**B**) as a function of solids sprayed during consecutive batches of spray drying on two different spray dryers. Circles represent collection on dryer 1 and crosses represent collection from dryer 2. The dashed line separates the two batches.

A comparison of in-process or running yields was conducted in the two spray drying experiments. Running wet yield during the spray drying batch is calculated as the ratio of the amount of wet powder collected to the amount of solids sprayed from the beginning of the batch up until the given time point in the batch and is expressed in percentage. Since the wet spray dried powder contains approximately 3–4.5% of solvent, the wet yield can exceed 100%. The in-process running wet solids collected against the amount of solids sprayed over two consecutive batches of spray drying is shown in Figure 3A. Translating the mass of wet solids to the running wet yield (as a function of the amount of solids sprayed) in Figure 3B, we find a noticeable difference in the wet yield between the two dryers for the second consecutive spray. In both dryers, the running yields have acceptable yields of >80% throughout the first batch. However, the second consecutive spray shows significantly lower product collection throughout the run in dryer 1 compared to the collection in dryer 2. As a result of the reduced net yield in dryer 1, nonstandard processing steps were performed to recover the wall deposits (To avoid significant wastage, in the middle of the second batch, solution spray was stopped and solvent was sprayed. An additional 15% of the batch was recovered. At the end of the batch, the additional gas flow was run to dislodge wall deposits, and 13% of the batch was recovered. Approximately 5% of the material was recovered from wiping the walls of the chamber. These are not included in the running wet yield.). While wall deposition was observed in dryer 2, these were typical of pharmaceutical spray drying with a build-up in the lower portions of the dryer. Wall deposition was not noticeable on the roof and the upper portion of the dryer.

Since dryer 2 did not show significant wall deposition and the resulting yield reduction, we focused our attention on the known differences between the two spray dryers and assessed each of our hypotheses systematically.

#### 3.2.2. Root Causes for Investigation

**Figure 4:**
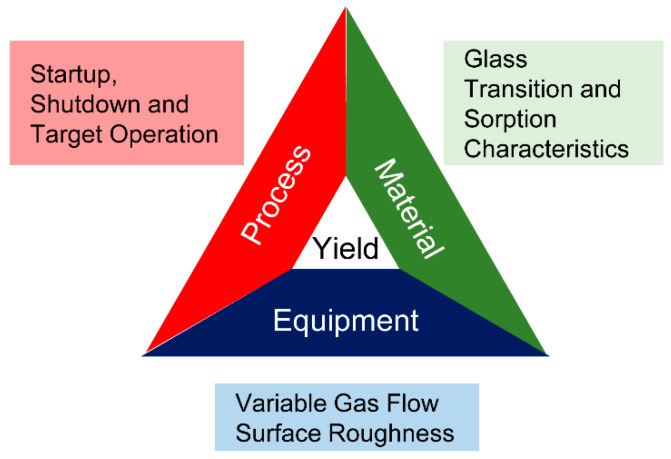
Root causes for yield reduction due to wall deposition.

An initial representation of the root causes for yield reduction based on our observations and our review of spray drying literature. We established three major categories: equipment, process, and material properties of the product, or an interplay of some or all these factors as shown in Figure 4. Based on the results of the repeatability test, we classified the known differences between dryer 1 and dryer 2 into targeted hypotheses within these categories to enable systematic investigation.

Equipment design elements, such as the surface roughness of the inner walls and gas disperser design could be responsible for the wall adhesion of partially dried spray-dried powder. Since gas disperser design is proprietary knowledge, the focus of the investigation was on surface roughness and gas flow variability.

The atypical wall deposition noted on the roof and upper walls of the drying chamber (Figure 2A–C) of a co-current spray dryer, raised concern that gas flow variability is a possible root cause. Yield reduction was noticed only during the second batch of a series of batches; therefore, the choice of shutdown and startup conditions was considered a potential factor.

Further, an understanding of the dependence of glass transition temperature on processing parameters such as outlet temperature and the concentration of the solvent in the drying gas could provide insight into factors leading to the adherence of spray-dried powder to the walls.

In our approach to understanding and overcoming yield reduction, we critically analyzed process parameters for unexpected behaviors and attempted to correlate process observations and our potential root causes. We will study testable hypotheses through targeted experiments at the laboratory scale when appropriate, and finally, implement a combination of our learnings on the spray dryer towards mitigating the yield reduction problem.

### 3.3. Equipment as Root Cause: Surface Finish on the Spray Dryer

The differences in the surface roughness of the two dryers were as follows: Dryer 1 has a specification for the inner chamber surface to be mechanically polished to ≤0.5 µm Ra followed by electropolishing, whereas Dryer 2 has no electropolishing, and chamber surface is specified to ≤0.8 µm Ra. The impact of surface roughness on the adherence of the spray-dried powder to the wall was studied using the angle of wall friction between the wall and the powder.

**Figure 5:**
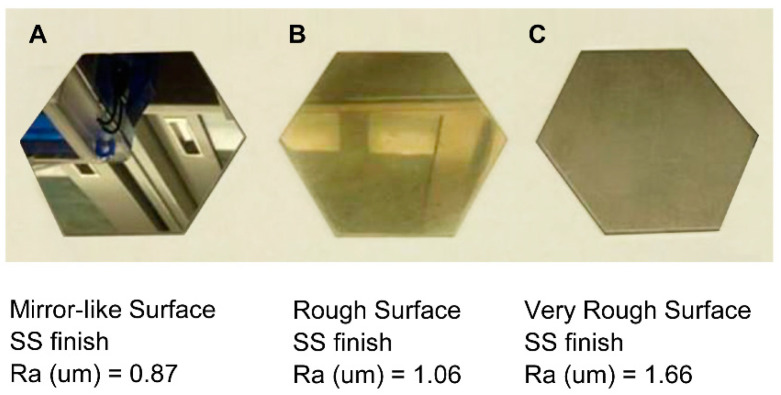
Stainless steel (SS) coupons with different surface finishes (**A**–**C**) used to assess trends in powder wall friction with an angle of incline measurements.

Powder wall friction experiments were conducted on three different stainless-steel coupons (shown in Figure 5) at 0.87 µm Ra with a mirror finish, 1.06 µm Ra, and 1.66 µm Ra without a mirror finish gave the angle of incline of 43.2° ± 1.8°, 32.4° ± 1.1°, and 28.7° ± 1.5°, respectively. While noting that the surface roughness used in powder wall friction experiments does not match the specifications of the two dryers, the trends in the measured angle of wall friction for the spray-dried powder inform us that smoother stainless-steel surfaces have a higher angle of powder wall friction. Therefore, while the experiments could not inform us if the difference in surface roughness of the inner walls of the dryer had a statistically significant difference in the angle of wall friction of the spray-dried powder, the trends inform us that the difference in surface roughness could serve as one of the contributing factors to wall deposition.

### 3.4. Equipment as Root Cause: Gas Flow Variability

During a typical operation of the spray drying process, the swirling gas flow patterns introduced into the spray dryer by the gas disperser results in drying the sprayed droplets and entrainment of the spray-dried powder to the cyclone with some wall deposition on the lower conical portion of the dryer. Due to the unexpected wall deposition of spray-dried powder on the roof and the upper cylindrical section of dryer 1 while operating at the same target conditions as dryer 2, a review of instrument data on gas flowrate, exhaust valve, and bag house filter differential pressure was assessed on a representative batch from the data historian on dryer 1.

**Figure 6:**
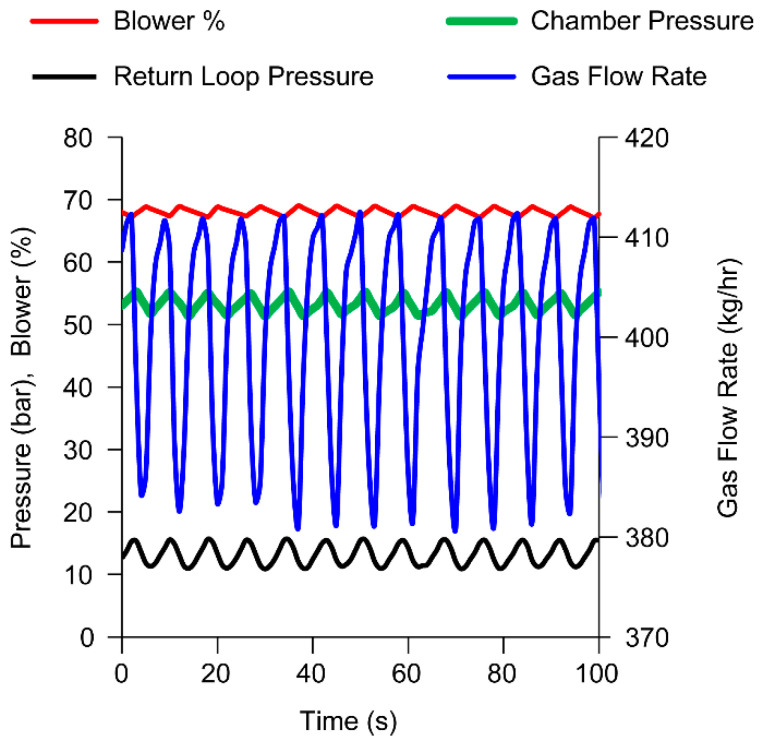
Gas flow rate variability during spray drying and Response of control loop during spray drying operation as shown by chamber pressure (green), return loop pressure (black), gas flow rate (blue), and blower % (red).

The results of the data analysis in Figure 6 showed that during the spray drying operation on dryer 1, an unexpected, periodic, and nearly sinusoidal variation in gas flow rate between ~380 to ~412 kg/h occurred when the set point was 400 kg/h. Since the closed cycle operation of the dryer utilizes a pressure control loop to maintain the level of nitrogen in the system, the control system monitored the variation of pressure on the return line (return loop pressure) from the spray dryer and responded by either adding nitrogen or opening an exhaust valve to maintain spray dryer chamber pressure. The pressure control loop was working in a cyclical mode with the exhaust valve opening and closing approximately 7.5 times a min modulating the percentage of blower capacity usage. This variation in gas flow rate and pressure gradients could disrupt the gas flow patterns in the spray dryer and potentially direct particle trajectories to the roof and upper walls of the spray dryer.

Potential sources of pressure variation are gas purges from the sealing shaft of the blower and the inline particle size analyzer, gas supply to differential pressure instruments on the cyclone and the baghouse filters, and pulsing of the bag house filters. One or a combination of these sources could trigger the pressure control loop to cause the observed gas flow variability. We found that modifications could not be made to the sealing shaft of the blower or the differential pressure instruments on the cyclone to ensure adequate operation of the dryer. Further, dryer 2, which does not have yield reduction issues, also does not have the inline particle size analyzer. Therefore, our approach to reducing the gas flow variability involved the removal of the inline particle size analyzer.

**Figure 7:**
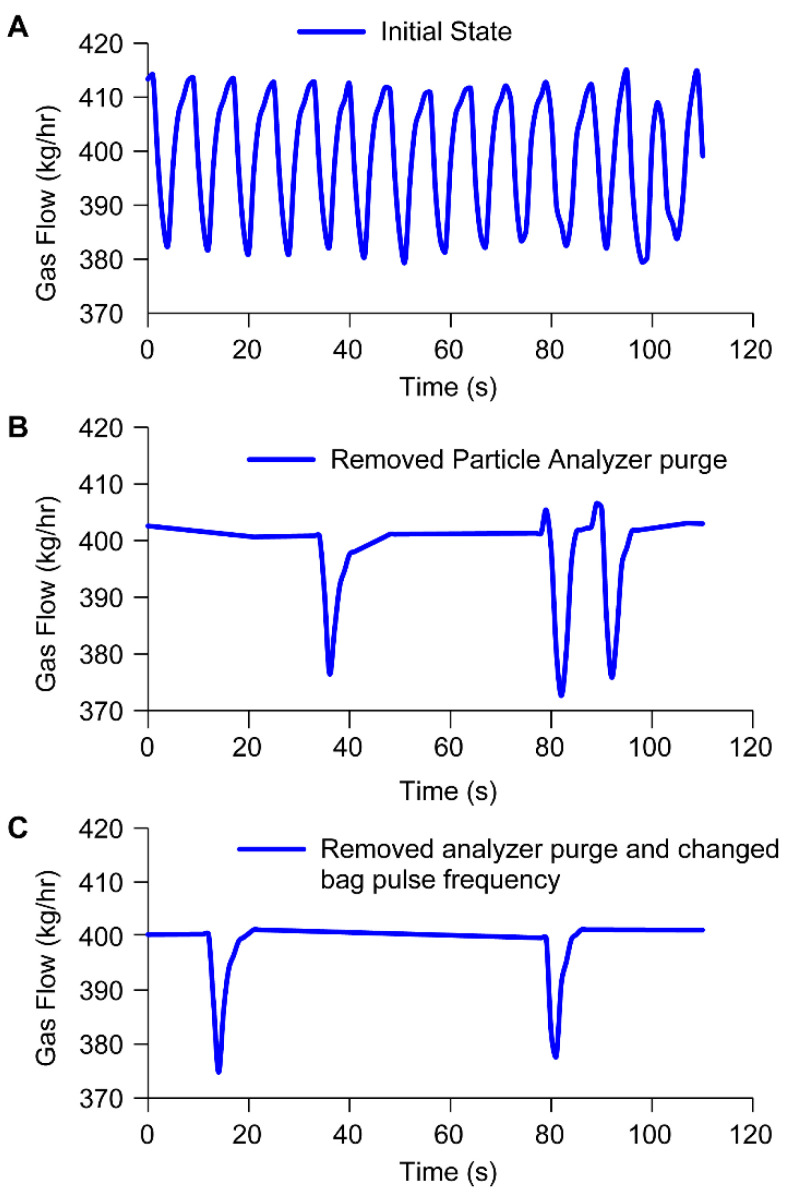
Mitigating gas flow variability on dryer 1: Gas flow vs. time in the base case (**A**) after removal of the inline particle size analyzer only (**B**) and after removal of inline particle size analyzer and change in Baghouse Filter Pulsing frequency (**C**).

The removal of the inline particle size analyzer and the associated nitrogen purge removed the high-frequency periodic (almost sinusoidal) variation in gas flow rate as seen in Figure 7B compared to Figure 7A. However, less frequent pulsing is noticeable in the gas flow rate-time graph, and the steep changes in flow rate are representative of exhaust valve motion when the valve is opened.

To further reduce this observed variability, we turned our attention to optimizing the pulsing operation of baghouse filters. The pulse frequency of the bag house filter was revised from 120 to 900 s thereby reducing the rate of nitrogen supply to the system for pulsing. The effect of this additional change on the gas flow rate-time graph is depicted in Figure 7C. The reduction in variability was quantified by evaluating the relative standard deviation. From the initial state (Figure 7A) to the removal of the inline purge (Figure 7B) coupled with a reduction in the pulsing frequency of the bag house filter (Figure 7C) reduced the relative standard deviation in gas flow rate from 2.8% to 1.2% to 0.8%. The corresponding reduction in the frequency of exhaust valve operation decreases from approximately 7.5 times a min (Figure 7A) to 0.3 times a min (Figure 7B) to 0.1 times a min (Figure 7C).

These approaches to reduce gas flow variability were implemented along with other mitigations from other root causes of the spray drying process in later sections and the results of the experiment are as discussed in Section 3.6 (overcoming yield reduction due to wall deposition). Briefly, a significant improvement in reducing the extent of wall deposits was observed in the upper part of the chamber walls and the roof. Only a slight dusting was noted compared to the extensive coverage without these changes.

### 3.5. Root Cause: Interplay of Material Properties and Process

While previous factors such as surface finish and gas flow variability could create conditions that support the wall deposition of the spray-dried powder, it is the material properties of the powder at specific process conditions that cause the powder adhesion to the wall. Therefore, to understand the stickiness of the powder, studies of glass transition temperature dependence on the level of spray-solvent in the powder and sorption studies were conducted. Thereafter, the operating conditions of the spray dryer were critically evaluated against this material property behavior to postulate potential root causes for the increased wall deposition during the second batch of spray drying.

**Figure 8:**
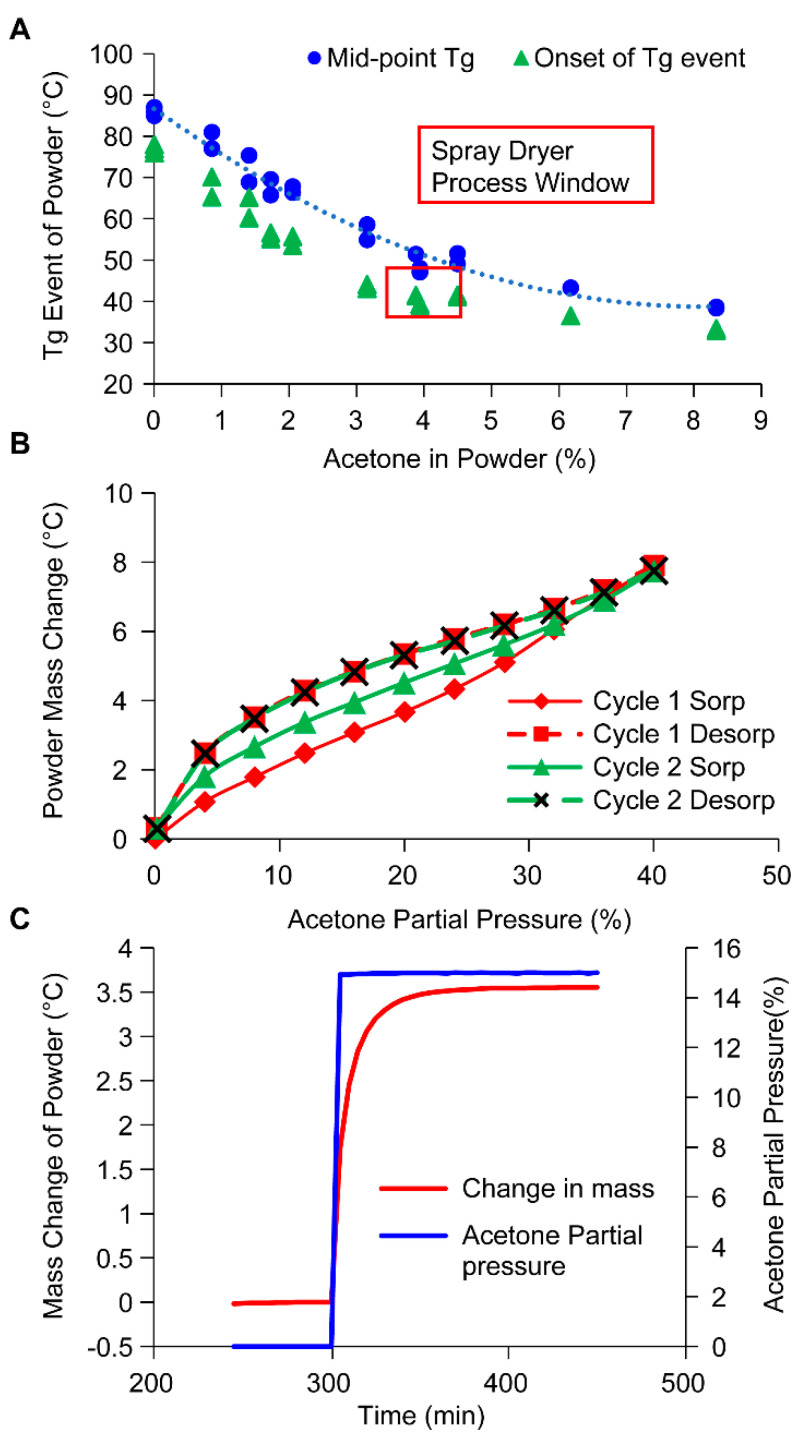
Material properties of spray dried powder: (**A**) Glass transition temperature as a function of acetone fraction in powder (% *w*/*w*). (**B**) Isothermal absorption and desorption of acetone by the spray dried powder at 25 °C in two cycles. Red diamonds and red squares represent absorption and desorption during the first cycle. Green triangles and black crosses represent absorption and desorption during the second cycle. (**C**) Kinetics of absorption of acetone by spray dried powder as measured by the percent change in mass of powder (red line) at 40 °C when subject to acetone partial pressure of 14.5% (blue line).

Modulated DSC experiments were conducted in hermetically sealed pans of spray-dried powder pre-equilibrated with different amounts of acetone. The glass transition of the spray dried powder was detected by heat capacity variations with temperature and reported as the onset of glass transition temperature (T_g, onset_) and mid-point glass transition temperature (T_g_), respectively. Figure 8A shows the dependence of T_g, onset,_ and T_g_ on the weight percent of acetone in the spray-dried powder. Independently, the amount of acetone in the wet spray dried powder when operating at the process outlet temperature range of 38–48 °C was measured to be 3.4–4.6% (*w*/*w*). As shown in the shaded region in Figure 8A, the process window overlaps with T_g, onset_ of the wet spray dried powder, and spans to the mid-point T_g_ line potentially making the powder prone to stick to walls.

Additionally, isothermal acetone sorption of spray-dried powder and the rate of sorption of acetone were studied experimentally using dynamic vapor sorption as shown in Figure 8B,C. The hysteresis in the change in the level of acetone (% by weight) during absorption (red solid line) and desorption steps (red dashed line) showed that a higher level of acetone is retained in the powder during the desorption process. A second cycle of sorption (green) resulted in a higher level of acetone retained by the powder compared to the first cycle. Further, when dried spray dried powder is exposed to 14.5% P/Po corresponding to the condition expected during a target closed loop operation of the spray dryer, very rapid uptake of acetone is observed raising concern about any undesired shifts in acetone content in the process gas. The combination of these studies shows that the spray-dried powder is prone to rapid uptake of solvent which plasticizes the powder. This plasticization could bring the powder close to its stickiness point temperature. The hysteresis in acetone sorption and desorption data suggests the material may be slow to release the acetone back during process shutdown or setpoint changes. Theoretically, these properties could lend the spray dried powder to a higher risk for sticky behavior and wall adhesion.

With an understanding of these material properties, and knowing the process parameters, we estimate the thermal history, and residual solvent concentration that wall-deposited powders are exposed to, in relation to glass transition behavior. The various steps of the operation per batch are (i) heat-up phase, (ii) solvent start-up phase, (iii) solution phase (spray dried powder is produced in this phase), (iv) solvent shutdown followed by (v) cool-down phase and (vi) gap period before the start of next batch.

Wall deposits are expected to be first formed during the solution phase (iii) when the first batch of the spray-dried solution is sprayed. Noting that the residual solvent concentration in the wet spray dried powder leaving the spray dryer is between 3.4–4.6% (*w*/*w*), as noted in Figure 8A, the process outlet temperature overlaps with the T_g, onset_ temperature of wet spray dried powder exiting the spray dryer. Therefore, the powders deposited on the wall are exposed to conditions that could cause it to stick to the wall. Switch from solution to solvent shutdown phase (iv) maintains a similar process temperature and partial pressure of solvent, and therefore no significant change in behavior is expected.

**Figure 9:**
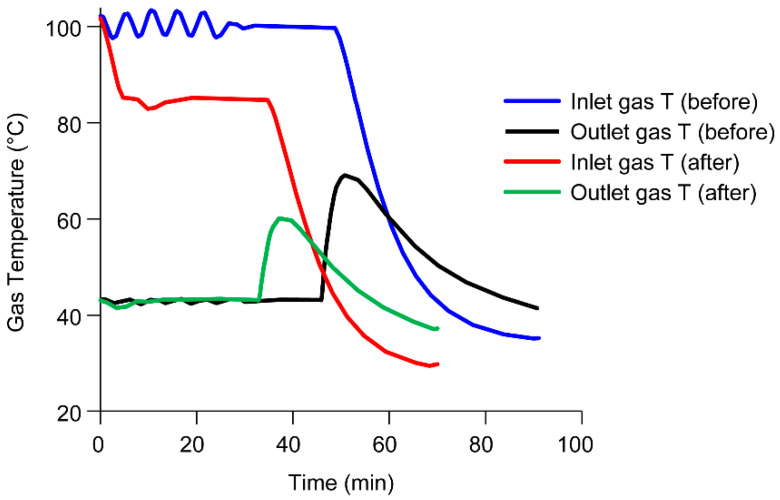
Interplay of material properties and processing: Inlet and outlet gas temperature batch data before and after modifications to the shutdown procedure.

In the cool-down phase (v) of the first batch, the solvent spray is turned off, and shortly afterwards, the inlet temperature is slowly ramped down as shown in Figure 9. Given the sudden loss in evaporation heat sink as a result of turning off the solvent spray, there is a short-term spike in outlet temperature up to 70 °C before slowly decreasing to room temperature. This happens because the inlet gas is heated by an electric heater. Electric heaters do not have a cooling function and the elements only cool by removal of heat from the flowing gas which is a slow process. Therefore, since heat removal from evaporation is no longer occurring, the outlet gas temperature increases briefly before ramping down. During these processes, outlet temperature spikes with almost no solvent present in the drying gas, the temperature of wall deposits could exceed T_g, onset_ and potentially increase the stickiness of the wall deposits. Process modifications to the cool-down step, (which is described later in Table 2), were successful in decreasing the extent of the outlet gas temperature spike and minimized thermal exposure of the wall deposits.

The next phase is the gap between two batches (vi), wherein the spray dryer remains shut down until the start of the next batch, and the spray dryer is not maintained in an inert state. Therefore, the wall deposits are equilibrated with room moisture. The presence of moisture could result in plasticization and reduction of T_g_ of the wall deposits. Thus, the spray, solvent shutdown, cool down and gap phases of the first batch seem to ensure that a small fraction of wall-deposited powders remain stuck to the wall even as we get an acceptable yield of 98% for this batch.

During the gas flow Heat-Up phase of the second batch, heated gas is blown through the drying chamber to warm up the equipment. The chamber is maintained at an outlet temperature of 65 °C for approximately 2 h, once again exposing the wall deposits from batch 1 to thermal conditions at or above T_g, onset_ and rendering the material more adhesive.

During the solvent startup phase of the second batch, the condenser set point changes from −5 °C to −15 °C at the same time when the solvent spray is initiated. Therefore, for a short 15–20 min until the condenser stabilizes to −15 °C, the inlet gas to the chamber carried a higher partial pressure of acetone leading to elevated acetone concentration in the resulting outlet gas. At 5 °C, thermodynamic modeling and estimation of saturation pressure via Antoine’s equation suggests the chamber outlet gas could go as high as 26% P/Po whereas it will drop to ~14% at steady-state operation at −15 °C. Acetone diffuses into the wall deposits, with the surface of wall deposits prone to higher concentrations of acetone and therefore further T_g_ reduction. These characteristics coupled with other root causes could make the wall deposits more likely to accumulate more particles during the solution spray phase of the second batch.

In contrast, dryer 2 with shorter start-up and short down periods, and different temperature control which led to fewer temperature spikes, may have avoided priming the wall deposits for future accumulation and therefore maintained the yields during the second batch. Thus, two mechanisms, exposure of wall deposits to temperatures higher than T_g, onset_ and exposure to conditions that lower the T_g_ of wall deposits were identified as root causes after critical assessment of the interplay of material properties and process conditions. The specific process conditions identified were specific to the long duration of the gas flow start-up and shutdown period, and specific settings to avoid temperature or acetone partial pressure spikes during start-up and shutdown. Additionally, we postulate that maintaining the inertness of the chamber between batches could also help avoid depression in the glass transition temperature of wall deposits.

### 3.6. Overcoming Yield Reduction Due to Wall Deposition

Based on our understanding of the root causes, and hypotheses leading to increased wall deposition, we assimilated the various mechanisms, namely gas flow stabilization, exposure of wall deposits to temperatures higher than T_g, onset_ and exposure to conditions that lower the T_g_ of wall deposits. Based on the root cause investigation, we identified process parameters to address the mechanism that impacts the yield. A summary of the mechanism and the process/equipment parameter to be revised are provided in Table 2. We conducted another experiment with multiple batches to implement all these factors, excluding any revisions to surface finish, to assess the change in yield behavior of dryer 1. 

**Table 2:** Summary of mechanism and process parameters to improve yield.

Target Mechanism	Parameter
Gas Flow Stabilization	Removal of Particle Size Analyzer and Associated Purge
	Increased cycle time between bag filter pulse event
Exposure to Temperatures above T_g, onset_	Minimize Inlet Temperatures during Heat Up Phase
	Minimize Heat Up duration during Heat Up phase
	Lower Inlet Temperature during cool-down phase of shutdown
T_g_ Suppression	Maintaining inert chamber between batches
	Condenser equilibrated to set point prior to solvent startup (at the end of Heat Up phase)

**Figure 10:**
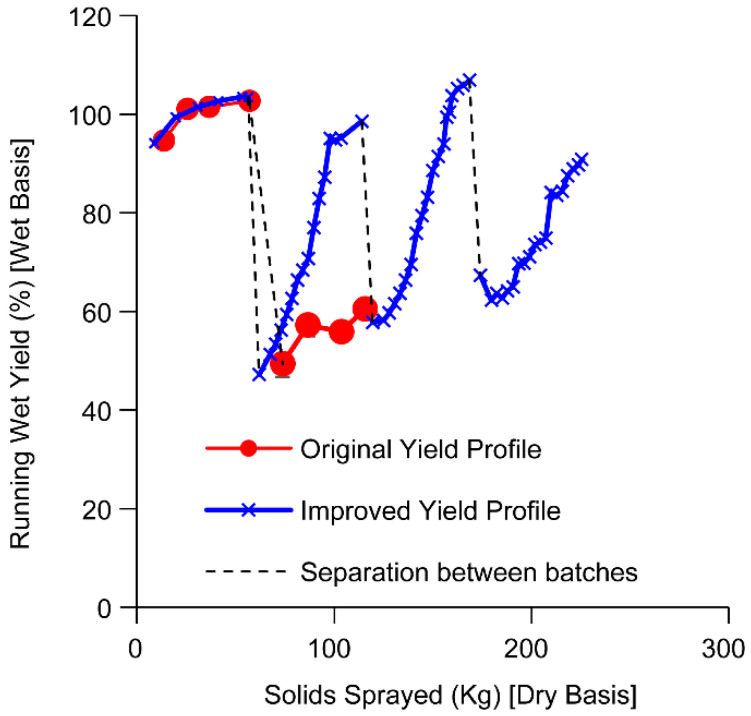
Improvement in yield for four consecutive batches on dryer 1. Closed circles (red) represent the original case when the yield of the second batch was reduced. Crosses (blue) represent the case after improvements were made to the process based on root cause investigation. Black dashed lines mark the end of a batch and the start of the next batch without any cleaning of the spray dryer.

The resulting yield improvement on a series of four consecutive batches is shown in Figure 10. The overall yield for each batch is greater than 90%. We note that wall deposition on the roof and upper portion of the chamber walls experienced slight dusting after the initial batch and it remains unchanged through the remaining batches. However, in dryer 1, the initial running yield of the second and other consecutive batches during the solution phase is still poor, but much more improved compared to the initial experiment indicating that wall adhesion is reduced but not eliminated. We note that while the impact of individual factors on the yield of the batch was not available, these improvements to the processing were also useful in ensuring that the product was not exposed to conditions that modify the physical characteristics of the powder.

Furthermore, this improvement was achieved without any significant changes to the target operation of the spray drying of the product. This approach was critical for pharmaceutical spray drying processing because if significant process changes to the target spray drying process are required then it could impact the performance of the tablet which contains the spray-dried powder. The impact of such changes, especially during later stages of drug product development may require additional studies to confirm the acceptability of such changes. In worst cases, such changes could impact the shelf life of the product and therefore, may not be feasible for the product.

A closer look at the running yield performance for the 4th batch indicates a marginal decrease in the final product yield compared to the previous batches. Thus, while the approaches identified above have been effective in mitigating yield reduction for a reasonable number of batches, the risk of wall deposition leading to reduced yields increases forlarger number of consecutively run batches. This behavior could be because, during the steady state operation of the dryer, the gas temperature and acetone partial pressure could bring the wall deposited spray dried powder close to the T_g, onset_ temperature. However, from a practical perspective, when 5–10 batches are required to be sprayed consecutively, it is common practice to scale up to the next larger scale to reduce the operational burden.

### 3.7. Recommendations for Overcoming Yield Issues during Different Stages of Development

Applying the learnings to the development of future amorphous solid dispersions especially during early stages of drug development, we highlight general and targeted approaches identified here to avoid the occurrence of such issues proactively. It is well-known that the process conditions of the spray drying process (such as the liquid: gas flow rate and process temperature) need to be chosen such that the product experiences temperatures below its wet glass transition temperature. It is also known through the review of the literature on sticking and wall deposition [7,9], that sticking often occurs at temperatures above the glass transition temperature. Studies have evaluated the rate and extent of wall deposition as a function of T-T_g_ [22]. However, occasionally for some drug and polymeric spray dried powders, operating at a temperature corresponding to the onset of the wet glass transition temperature (despite being below T_g_) may exhibit atypically strong adhesion to the wall leading to reduced yield and delayed recovery.

Therefore, during the early development of the spray drying process, characterization of the dependence of wet glass transition temperature (T_g_ as well as T_g, onset_) on the residual solvent levels relative to the operating conditions are critical. If the intended processing condition overlaps with wet T_g,onset_ region as such in Figure 8A, then there are several approaches listed below that may be proactively applied to avoid the occurrence of risk [23].

A process-centric approach involves moving away from and below the wet T_g, onset_ curve (Figure 8A) by reducing the liquid: gas flow rate and/or process outlet temperature (T). Such a process space can be estimated using a simple energy balance model of the spray dryer. Such approaches are robust if complemented by experimental confirmation of yield robustness over a wider range of the liquid: gas flow rates and processing temperatures in addition to the impact on the attributes of the resulting spray-dried powder on the final drug product. Attention to the start-up and shutdown procedures should be paid to avoid conditions that cause depression in the wet glass transition behavior and cause depression of the wet glass transition behavior and exposure of wall deposited powder to a temperature above the glass transition temperature.

Formulation-centric approaches include a selection of polymeric stabilizers or modification of the ratio of drug to polymer to ensure that the resulting amorphous solid dispersion has sufficiently high T-T_g, onset_. Modification of solvent systems could also be considered if the new solvents can adequately modify the glass transition temperature dependence on the residual solvent level of the new solvents such that T-T_g, onset_ is high. Modification of solvent systems could also be considered if the new solvents can modify the glass transition temperature dependence on the residual solvent level of the new solvents.

When applying our learnings to the occurrence of reduced yield behaviors later in drug development as in our case, we recommend an assessment of the mechanisms. At the first observation of yield reduction, it is important to confirm or rule out common failure modes leading to wall deposition, such as atypical or incomplete or significant differences of atomization arising from nozzle variability or poor droplet drying leading to agglomeration on walls. By comparing the attributes of wall-deposited material against the steady-state material, we confirmed that these failure modes were not contributing to this specific problem as significantly larger and agglomerated particles would be expected and associated potential shifts in bulk density properties. If the attributes are different [24], a thorough assessment of attributes can be used to identify root causes such as the sensitivity of atomization and/or the interplay with the gas flow in the drying chamber.

If the mechanisms point to the interplay of glass transition thermodynamics with processing space, employing practical approaches demonstrated here to avoid conditions prone to depression of glass transition temperature and exposures to a temperature above glass transition temperature could be useful. It is important to note that the changes to start and shutdown conditions in addition to a reduction of gas flow variability arising from inline analytical equipment were adequate to improve the yield in our case because the overall target processing was not at or above the wet glass transition temperature of the powder. We also note that although this study has been effective in mitigating yield reduction for a few batches, wall-build up can impact yields when running a longer number of batches. Therefore, if the implementation of these changes does not result in significant improvement in yield, then approaches such as process or formulation changes proposed for the early stage of drug development may be necessary.

Further, during the technical transfer of the spray drying process to different spray drying units, a critical assessment of gas flow variability must be conducted in addition to other process fit considerations. In our case, while the variations in gas flow were noted, they were attributed to normal process variability until a targeted in-depth understanding of the gas flow and the feedback control clarified that the source of near periodic variability was due to the presence of the inline analytical equipment. Therefore, a thorough assessment studying the impact of additional inline equipment on the gas flow variability during qualification is important for the successful operation of the spray drying.

## 4. Conclusions

An atypical yield issue during pharmaceutical spray drying is described and practical solutions to effectively mitigate the wall deposition-related yield issues are demonstrated in this paper. After identification of the yield reduction problem, moving the target process further away from and below the onset of glass transition of the powder through simple mass and energy balance in the drier is a simple, useful, and possibly common solution. However, noting that dryer 2 can be operated under similar conditions without yield reduction, this investigation puts a spotlight on hypothesis-based understanding of the driving forces for the reduction in yield and translating into approaches to overcome the yield reduction problem on dryer 1. While in-depth understanding requires additional experimentation varying these factors one at a time, the evidence of improvement in yields offers strength to these reasoned and mechanism-based solutions.

As with most real-world problems, our root causes revealed more than one factor that could be contributing to the wall deposition. Gas flow variability introduced by the use of an inline particle size analyzer resulted in partially dried particles being forced to the roof and upper wall regions of the drying chamber. Start-up and shutdown conditions that exposed wall-deposited material to temperatures near or above the onset of glass transition, and conditions that suppressed its glass transition temperature coupled with the gas flow variability increased the extent of wall deposition during the subsequent batches. Incorporating changes that addressed these root causes reduced the extent of wall deposition and improved the recovery rate of re-entrained powder during production.

Understanding the gas flow variability due to purges and pulsing using computational fluid dynamics of transient flow in the spray-dryer could be considered to further optimize the performance of the spray dryer. The internal chamber surface finish remains an interesting factor for further research to evaluate as a separate factor. However, predictive models of stickiness and wall deposition in a spray drying operation are very difficult since the dynamics of sticking are more complex than what glass transition theory and computational fluid dynamics can offer [23].

The root cause analysis presented in the paper illustrates a hypothesis-driven approach to troubleshooting a complex problem such as yield issue during spray drying. We have further distilled our learnings into recommendations for early and late-stage drug development approaches. In addition, we have highlighted the importance of a thorough assessment of process parameters such as gas flow variability during the implementation of in-line analytical equipment in the spray dryer during qualification. Such assessments would enable seamless technical transfer of processes between different dryers. We hope this work provides useful ideas and concepts for process engineers to study when dealing with a yield challenge in their spray drying process.

## Figures and Tables

**Table 1:** Comparison of wall-deposited powder vs. powder collected in the exit of the spray dryer.

Location	Particle Size Distribution (d_10_, d_50_, d_90_ µm)	Bulk/Tap Density (g/cm^3^)	Glass Transition Temperature, T_g_ (°C)
Wall deposited material	24, 46, 90	0.23, 0.35	85
Steady-state run	18, 40, 86	0.22, 0.37	86

## Data Availability

Datasets used as a part of these studies are proprietary under Merck & Co., Inc. Redacted datasets are available upon request through the corresponding author.

## Supplementary Materials

The following supporting information can be downloaded at: https://www.mdpi.com/article/10.3390/pharmaceutics15082137/s1.
