# Supplementary material for: Driving Spray Drying towards Better Yield: Tackling a Problem That Sticks Around
Source: Pharmaceutics. 2023 Aug 14;15(8):2137. doi: 10.3390/pharmaceutics15082137 (PMC10459734; doi:10.3390/pharmaceutics15082137)
Supplement: Supplementary file 1 [file pharmaceutics-15-02137-s001.zip › pharmaceutics-2506045-supplementary.pdf]

Supplementary Information

Several different material characterization techniques have been used to assess the spray dried powder as well as in the investigation of the root causes. The details of the characterization are provided.

1.1. Scanning Electron Microscopy:

Sample morphology is assessed with a FEI Quanta FEG 250 environmental scanning electron microscope (ESEM). Each sample is mounted onto an aluminum stub using double-sided carbon tape. The microscope is operated at 20 kV, spot size of 3.0, ~0.8 torr chamber pressure, and ~10 mm working distance in Secondary Electron imaging mode.

1.2. Particle Size Distribution (PSD):

PSD after secondary drying was measured using a Microtrac S3500 laser diffraction particle size analyzer with flow rate set at 70% without sonication. IsoparG with 0.25% lecithin (w/v) was used as the carrier fluid. The instrument parameters were set as transparent, irregular particles with a refractive index of 1.51, fluid refractive index of 1.42, and geometric 8 root progression between 0.0215 and 1408 μm, with residuals disabled. Standard distributions are applied, and results are reported as volume distributions as averages for duplicate sample measurements.

1.3. Bulk/Tap Density:

Powder was added into a tared 100 mL graduated glass cylinder (class A) (using a stainless-steel funnel to minimize effects on powder compaction) to 80-100% cylinder fill. The initial volume and the weight of the sample were used to calculate the bulk density. The cylinder was tapped 1250 times on a Vankel Tapped Density Tester until the volume of the sample was constant within 1 mL. The constant final volume was used to determine tapped density.

1.4. Gas Chromatography

The Headspace-Gas Chromatography (HS-GC) method for determining Residual Solvents used a 7890B series Agilent GC system with FID and a 7697A Agilent headspace autosampler. Agilent DB-624 capillary column, 30 m × 0.32 mm inner diameter and 1.8 μm film thickness was used. GC conditions used were Helium carrier gas flow rate of 2.0 ml/min, split mode of 100:1, inlet & detector temperature of 240 °C, initial oven temperature of 40 °C with a 3-minute hold time before ramping at 25 °C/min to 240 °C and held for 3 minutes.

HS conditions used were oven temperature of 135 °C, shaker set at high for 15 minutes, loop temperature of 165 °C, transfer line temperature of 170 °C, pressurization time of 0.1-minute, loop equilibration time of 0.15 minute, injection time of 1.0 minute and vial pressure of 20 psi. The diluent used was N-Methyl-2-pyrrolidone. Samples were prepared at concentration of 40mg/mL (4 mL NMP)/ 20mL HSGC vial and quantified using 0.02% v/v solvent standard.

1.5. Dynamic Vapor Sorption (DVS):

DVS is measured with a DVS Resolution by Surface Measurement System equipped with ultra-balance and stand housed in a precisely controlled constant-temperature incubator. Acetone partial pressure is generated by mixing dry and saturate nitrogen with the closed loop control measured by Speed of Sound sensor. The equilibrium of the sample mass is set at 0.001% change in mass in 10 minutes and maximum 3 hours. Isotherm is plotted at the equilibrium point of each set acetone partial pressure.

1.6. Powder Wall Friction – Angle of Incline Measurement:

The powder and a ramp setup whose angle of incline can be changed and measured are placed inside glove box equilibrated to 30%± 5 %RH at ambient temperature. To measure the angle at which powder no longer adheres to surface a stainless-steel coupon of varying surface finish (0.87, 1.06 or 1.66 µm), is placed on the ramp of a protractor at an incline angle of 0 degrees. On top of the stainless-steel coupon a compact of spray dried powder (formed by placing approximately 9g of powder in a 6 mm diameter x 1 mm thick ring with a 118g weight placed atop) is then placed. The angle of the incline is measured as the angle when the powder bed begins to move as the ramp angle is incrementally increased.

1.7. Differential Scanning Calorimetry

Thermoanalysis was conducted on a TA Instruments DSC (*Differential Scanning Calorimetry)* Q2500 with Tzero hermetic lid and pan (TA Instruments). The sample is equilibrated at -20°C, heated at 2 °C/minute to 230 °C with 1.5 °C/60.0 second modulation. The glass transition (T_g) midpoint and onset are analyzed on the reverse heat flow using TA Instruments Trios (5.1.1.46572) software.

Different weight content of acetone is diffused through gas phase into DSC and GC samples in a seal oven-GC vial. Due to fast acetone loss from powder sample during sample transferring and DSC pan crimping, DSC samples (8-12 mg) and GC samples (approx. 170 mg total in 5 compacts) are compressed into 6/32 round flat-faced compacts at 50 kgf. For each level of

acetone in the same oven-GC vial, the DSC and GC samples are equilibrated for 4 hours after an estimated amount of acetone is injected. The samples are immediate sealed in a pre-weighed oven GC vial for GC analysis and crimped in pre-weighed DSC pan/lid sets for modulated DSC analysis.
